# Correlation of the Six-Minute Walk Test, Diffusing Capacity for Carbon Monoxide, and Pulmonary Function Parameters in Patients With Interstitial Lung Disease: A Cross-Sectional Study

**DOI:** 10.7759/cureus.94917

**Published:** 2025-10-19

**Authors:** Sushant Muley, Sushant Meshram, Avinash Gandhare, Ravichandra Konjengbam, Srikanth Bhaisare

**Affiliations:** 1 Department of Pulmonary Medicine, Government Medical College, Nagpur, Nagpur, IND

**Keywords:** 6-minute walk test, dlco, fibrosis, fvc, interstitial lung disease, pulmonary function test

## Abstract

Background

Interstitial lung disease (ILD) encompasses a broad group of progressive lung disorders marked by inflammation and fibrosis, leading to irreversible damage. Early screening plays a pivotal role in identifying disease onset and optimizing patient outcomes. This study assessed the correlation of diffusing capacity for carbon monoxide (DLCO) with the six-minute walk test (6-MWT) and pulmonary function test (PFT) parameters. Moreover, the correlation between 6-MWT and PFT parameters was evaluated.

Methods

This cross-sectional study, involving 30 patients with high-resolution computed tomography-proven ILD, was performed over a period of 24 months (October 2022 to September 2024) in the Department of Respiratory Medicine of Government Medical College, Nagpur, in Nagpur, India. All patients underwent 6-MWT and PFT. Vital parameters were assessed before and immediately after 6-MWT, and the six-minute walk distance (6-MWD) was calculated.

Results

6-MWT had a weakly positive and significant correlation with forced vital capacity (FVC) (r=0.38; p=0.037), but not with forced expiratory volume in one second (FEV1) and DLCO (p>0.05). DLCO had a positive and non-significant correlation with total lung capacity, FEV1, and FVC, while correlation with FEV1/FVC ratio was negative and non-significant (p>0.05). Actual 6-MWD was significantly lower than the predicted value (p<0.001). Immediately after 6-MWT, there was a significant decrease in oxygen saturation, while systolic blood pressure, pulse rate, and respiratory rate increased significantly compared to baseline 6-MWT values (p<0.001), with diastolic blood pressure being comparable at both intervals (p>0.05).

Conclusion

6-MWT had a weak but significant correlation with FVC, while the correlation between other parameters was not significant. These tests highlight functional limitations and physiological derangements, thus strengthening their role in ILD management.

## Introduction

Interstitial lung disease (ILD) includes a wide spectrum of lung conditions that are associated with inflammation, progressive fibrosis, and architectural distortion of the pulmonary interstitium. These changes hamper gas exchange and lead to restrictive lung physiology [1,2]. ILDs, including idiopathic pulmonary fibrosis (IPF), connective tissue disease-associated ILD, and hypersensitivity pneumonitis, are responsible for a substantial burden of global morbidity and mortality attributed to respiratory disorders [3]. The global prevalence of ILD varies widely from 6.3 to 76 per 100,000 individuals, with IPF being the predominant idiopathic subtype [4]. From the Indian perspective, limited epidemiological data suggest a rise in ILD burden with a high mortality rate (around 46% at four years) [5], especially among those with a rural background owing to occupational exposures and use of biomass fuel; however, precise estimates still remain underreported [6].

A multidisciplinary approach is required to diagnose ILD, including clinical examination, pulmonary function tests (PFTs), high-resolution computed tomography (HRCT) of the chest, and histopathological examination, if required [7]. Among various PFT parameters, lung volumes (e.g., forced vital capacity (FVC)) suggest restrictive pathology, while diffusing capacity for carbon monoxide (DLCO) is a critical indicator of disease severity, highlighting the impairment of gas exchange [8]. In ILD, the six-minute walk test (6-MWT) is a simple tool that allows functional assessment and correlates with disease progression as well as mortality [9]. However, the correlation between DLCO, 6-MWT, and lung volumes is underexplored, especially in Indian patients with ILD, where genetic and environmental factors may affect disease behavior.

In ILD, some of the authors have reported weak to moderate significant correlations between DLCO and 6-MWT [10,11], while others have reported no significant correlation between them [12]. Moreover, PFT parameters correlate significantly with 6-MWT [11,13]. However, this association is not consistent with DLCO [14,15]. Thus, the precise correlation between these parameters remains to be determined, especially in Indian patients with ILD. To address this critical knowledge gap, the present study assessed the correlation of DLCO with 6-MWT and PFT parameters in patients with ILD. The secondary objective was to evaluate the correlation between 6-MWT and PFT parameters.

## Materials and methods

Study design and ethics

This cross-sectional observational study was performed over a period of 18 months (September 2023 to February 2025) in the Department of Respiratory Medicine of Government Medical College, Nagpur, in Nagpur, India. The study was approved by the Institutional Ethics Committee of Government Medical College, Nagpur (approval number: MUHS/PG/E-1/1501/1062/09), and written informed consent was taken from all the patients prior to study initiation.

Study participants

The study included 30 adult patients, aged 18 years or more, of either sex, with a confirmed diagnosis of ILD based on characteristic HRCT findings of honeycombing, usual interstitial pneumonia (UIP) pattern, non-specific interstitial pneumonia (NSIP) pattern, and/or connective tissue disease-associated ILD, in a stable clinical condition, with no recent exacerbations or acute worsening of their ILD within the last 4-8 weeks, and who were physically capable of performing 6-MWT without contraindications, such as unstable angina, recent myocardial infarction, or musculoskeletal conditions that limits ability to walk. Exacerbation was defined as worsening of respiratory symptoms like cough, breathlessness, and hypoxia requiring hospital admission. In contrast, patients with oxygen saturation <88% on room air who are unable to safely perform the 6-MWT without supplemental oxygen, co-existing obstructive lung diseases such as chronic obstructive pulmonary disease and asthma, unstable cardiac conditions such as recent myocardial infarction, uncontrolled arrhythmias, and severe heart failure, and severe cognitive impairment or psychiatric conditions were excluded.

Study procedure

Patients were recruited from the outpatient department and wards of the Department of Respiratory Medicine. Following enrollment, all patients underwent 6-MWT, two-dimensional echocardiography (2D-ECHO), chest HRCT, and PFTs. The 6-MWT was performed as per the American Thoracic Society (ATS) recommendations [16]. To allow reproducibility, all tests were performed by a single investigator in a 30-m-long hall, using standard encouragement, and the six-minute walk distance (6-MWD) was recorded. Additionally, pulse rate (PR), respiratory rate (RR), systolic blood pressure (SBP), diastolic blood pressure (DBP), and oxygen saturation (SpO2) were recorded before and immediately after 6-MWT. HRCT images were analyzed, and patterns of pulmonary changes attributed to ILD were studied. PFTs were performed by a trained technician, with 10 years of experience, using the Jaeger Master Screen PFT system (Germany) as per the ATS guidelines. All test procedures conformed to the acceptability and repeatability criteria [17], and the findings included FVC, total lung capacity (TLC), forced expiratory volume in one second (FEV1), and DLCO. DLCO was evaluated with the single-breath method. The percentages of predicted values of PFT parameters were used. Moreover, 2D-ECHO was performed to rule out pulmonary arterial hypertension, which may be associated with ILD on presentation.

Sample size

Based on the study by Muhammed Shafeeq et al. [18], the prevalence of IPF was 38.6%. Considering the Z-value of 1.96 at 95% CI and allowable error of 0.175, the sample size was calculated to be 30.

Statistical analysis

The data was analyzed with IBM SPSS Statistics for Windows, Version 23.0 (IBM Corp., Armonk, New York, United States). The categorical and continuous variables are represented as frequency (percentage) and mean (standard deviation), respectively. The Pearson correlation coefficient was used to assess the correlation between various continuous variables (DLCO, 6-MWT, and lung volumes). Paired t-test was used to assess the association between various continuous variables (6-MWD, PR, RR, SBP, DBP, and SpO2). A two-tailed probability (p) value of <0.05 was considered statistically significant.

## Results

The patients were slightly male predominant (53%) with a mean age of 61.0±12.0 years. The most frequent presenting symptoms were breathlessness (86%) and dry cough (66.6%). The mean body mass index (BMI) and Modified Medical Research Council (MMRC) grade were 21.3±4.7 kg/m^2^ and 2.1±0.7, respectively. Of the total 20 patients, 12 (40%) were active smokers, while three (10%) were exposed to chulha smoke. The majority of the patients were housewives (36.7%) and farmers (30%) (Table 1).

**Table 1:** Demographic and clinical characteristics BMI: body mass index; MMRC: Modified Medical Research Council

Characteristics	n=30
Age, years, mean±SD	61.0±12.0
Gender, n (%)	
Female	33 (47%)
Male	16 (53%)
Symptoms, n (%)	
Breathlessness	30 (86%)
Dry cough	20 (66.6%)
Cough with scanty expectoration	4 (13.3%)
Chest pain	3 (10%)
Joint pain	2 (6.7%)
BMI, kg/m^2^, mean±SD	21.3±4.7
MMRC grade, mean±SD	2.1±0.7
Smoker, n (%)	12 (40%)
Chulha, n (%)	3 (10%)
Occupation, n (%)	
Housewife	11 (36.7%)
Farmer	9 (30%)
Saxophone	1 (3.3%)
Detonator machine maintenance	1 (3.3%)
Nurse	1 (3.3%)
Attendant in hospital	1 (3.3%)
Brick factory	1 (3.3%)
Police officer	1 (3.3%)
Tile setter, exposure to cement	1 (3.3%)
Truck driver	1 (3.3%)
Alcohol seller, floor tile fitter	1 (3.3%)
Builder, cement exposure	1 (3.3%)

On HRCT of the chest, the most common findings were UIP pattern (63.3%) and NSIP pattern (26.7%). The 2D-ECHO findings were mainly normal (46.7%) and mild tricuspid regurgitation (33.3%) (Table 2).

**Table 2:** Radiological and echocardiographic findings HRCT: high-resolution computed tomography; UIP: usual interstitial pneumonia; NSIP: non-specific interstitial pneumonia; G1DD: grade 1 diastolic dysfunction; TR: tricuspid regurgitation; MR: mitral regurgitation; AR: aortic regurgitation; LVH: left ventricular hypertrophy; PAH: pulmonary arterial hypertension; *ground-glass opacities, interstitial thickening, and volume loss

Findings	n=30
HRCT, n (%)	
UIP pattern	19 (63.3%)
NSIP pattern	8 (26.7%)
UIP with honeycombing	5 (16.7%)
UIP with traction bronchiectasis	2 (6.7%)
Fibrotic NSIP	1 (3.3%)
Other*	7 (23.3%)
2D-ECHO, n (%)	
Normal	14 (46.7%)
Mild TR	10 (33.3%)
G1DD	4 (13.3%)
Mild MR	4 (13.3%)
LVH	2 (6.7%)
PAH	2 (6.7%)
Mild AR	1 (3.3%)

On pulmonary function test, most of the patients had FVC <50% (66.7%), FEV1/FVC ratio >70 (83.3%), and DLCO <40% (56.7%) (Table 3).

**Table 3:** Pulmonary function test parameters DLCO: diffusing capacity for carbon monoxide; FVC: forced vital capacity; FEV1: forced expiratory volume in one second; ILD: interstitial lung disease

Parameters	n=30
FVC, %, n (%)	
<50	20 (66.7%)
50-60	4 (13.3%)
60-80	5 (16.6%)
>80	1 (3.3%)
FEV1/FVC ratio, n (%)	
>70	25 (83.3%)
≤70	5 (16.7%)
DLCO category, %, n (%)	
<40	17 (56.7%)
41-60	6 (20%)
61-75	2 (6.7%)
76-140	3 (10%)
>140	2 (6.7%)

6-MWT had a weakly positive correlation with FVC (r=0.38) and FEV1 (r=0.34). Though the correlation of 6-MWT with FVC was statistically significant (p=0.037), there was a trend towards significance with FEV1 (p=0.067). DLCO had a positive correlation with 6-MWT, TLC, FEV1, and FVC, while the correlation between DLCO and FEV1/FVC ratio was negative. Moreover, the correlation of DLCO with all these parameters was not statistically significant (p>0.05) (Table 4).

**Table 4:** Correlation between DLCO, 6-MWT, and lung volumes r: Pearson correlation coefficient; DLCO: diffusing capacity for carbon monoxide; TLC: total lung capacity; 6-MWT: six-minute walk test; FVC: forced vital capacity; FEV1: forced expiratory volume in one second

Characteristics	r	p
6-MWT vs. FVC	0.38	0.037
6-MWT vs. FEV1	0.34	0.067
DLCO (%) vs. 6-MWT	0.21	0.273
DLCO (%) vs. TLC	0.26	0.160
DLCO (%) vs. FEV1	0.06	0.762
DLCO (%) vs. FVC	0.01	0.970
DLCO (%) vs. FEV1/FVC ratio	-0.16	0.316

The actual 6-MWD among the patients with ILD was significantly lower than the predicted 6-MWD (p<0.001) (Table 5).

**Table 5:** Actual versus predicted 6-MWD 6-MWD: six-minute walk distance

6-MWD	Actual (n=30)	Predicted (n=30)	p
Mean±SD	359.5±121.1	509.7±84.8	<0.001

Immediately after 6-MWT, there was a significant decrease in SpO2 (p<0.001), while SBP (p<0.001), PR (p<0.001), and RR (p<0.001) increased significantly compared to baseline 6-MWT values. However, DBP was comparable between baseline and immediately after 6-MWT (Table 6).

**Table 6:** Pre- and post-6-MWT vital parameters 6-MWT: six-minute walk test; SPO2: oxygen saturation; SBP: systolic blood pressure; DBP: diastolic blood pressure; PR: pulse rate; RR: respiratory rate

Vital parameter	Pre-6-MWT	Post-6-MWT	p
SPO2	93.7±2.4	87.9±3.3	<0.001
SBP	119.3±8.3	126.3±9.1	<0.001
DBP	79.3±5.2	79.7±4.9	0.573
PR	109.9±12.7	123.4±10.1	<0.001
RR	22.8±1.9	31.1±2.9	<0.001

## Discussion

In patients with ILD, 6-MWT is a simple, frequently used, and repeatable assessment tool for the assessment of functional capacity. The present study evaluated the correlation of DLCO with 6-MWT and PFT parameters as well as the correlation between 6-MWT and PFT parameters. Moreover, physiological responses before and after 6-MWT were assessed. The principal findings suggested a weak but statistically significant positive correlation between 6-MWT and FVC, while the correlation with FEV1 was suggestive of a trend towards significance. Moreover, DLCO had positive but non-significant correlations with TLC, FEV1, FVC, and 6-MWT, while a negative correlation was observed with the FEV1/FVC ratio. Additionally, assessments of vital parameters after 6-MWT revealed a significant reduction in SpO2, along with significant increases in SBP, PR, and RR, while DBP remained unchanged.

The findings on PFT revealed severe restrictive impairment, with FVC <50% and DLCO <40% observed in 66.7% and 56.7% of patients, respectively. The restrictive ventilatory defect characteristic of ILD was confirmed with a preserved FEV1/FVC ratio >70 observed in 83.3% of patients. The weak positive correlation between 6-MWT and FVC is consistent with the available literature, suggesting that restrictive pulmonary physiology in ILD leads to a decline in exercise capacity. FVC, an indicator of the severity of restrictive lung disease, suggests lung volume restriction, which may hinder oxygen uptake and stamina during walking [19]. The significant correlation between 6-MWT and FVC reinforces the belief that lung volume restriction plays a critical role in functional impairment. However, weak correlation highlights that other factors, including pulmonary vascular disease, musculoskeletal disease, or deconditioning, may also affect 6-MWT [20,21].

The correlation between 6-MWT and FEV1 revealed a trend towards significance, suggesting that ILD mainly affects lung parenchyma and not airways, making FEV1 a less sensitive indicator of disease severity relative to FVC [8]. Nonetheless, fibrotic lung diseases have some degree of airflow limitation [22], thereby explaining the observed correlation.

We also observed a positive correlation of DLCO with 6-MWT, TLC, FEV1, and FVC, though it was not statistically significant. This finding is in agreement with available literature suggesting that DLCO is a marker of gas exchange efficiency, which is impaired in patients with ILD due to the thickening of the alveolar-capillary membrane and pulmonary fibrosis [8]. The lack of significant correlation may be attributed to the small sample size or the heterogeneous nature of ILD subtypes, including hypersensitivity pneumonitis, respiratory bronchiolitis-associated ILD, and connective tissue disease-related ILD (CTDILD), where impairment of DLCO varies widely [23]. The restrictive rather than obstructive nature of ILD is further supported by a negative correlation between DLCO and FEV1/FVC ratio, as increased FEV1/FVC ratio is characteristic of restrictive diseases [8].

The significant reduction in actual 6-MWD relative to predicted values highlights functional limitation due to ILD. This finding is consistent with various studies that have reported decreased exercise tolerance in patients with ILD due to hypoxemia, ventilatory constraints, and decreased cardiac output secondary to pulmonary hypertension [19,24]. The difference between actual and predicted 6-MWD emphasizes the clinical utility of the 6-MWT in assessing the impact of disease beyond standard PFTs [25].

A significant reduction in SpO2 observed after 6-MWT is concordant with exercise-induced hypoxemia due to lung fibrosis-associated impaired oxygen diffusion and ventilation-perfusion mismatch [8,19]. The significant increase in SBP, PR, and RR suggests the respiratory and cardiovascular adaptations to increased metabolic demand that occur during and immediately after exercise [26], while the lack of significant change in DBP after 6-MWT indicates stable peripheral vascular resistance, owing to compensatory vasodilation while performing physical exercise [27].

The findings of the present study emphasize that while PFT parameters (FVC, FEV1, and DLCO) provide insight into the severity of ILD, they only partially explain exercise limitations in this group of patients. The 6-MWT provides additional prognostic value by incorporating cardiopulmonary and musculoskeletal responses, thus making it a useful tool for monitoring the progression of disease and functional assessment [8]. The significant decline in SpO2 after 6-MWT also supports the need for evaluation of supplemental oxygen in patients with ILD who have exertional hypoxemia [28].

The present study had certain limitations. First, a small sample size limits the generalizability of the findings as it does not represent the wide spectrum of the ILD population with varying etiologies and severities. Second, lack of follow-up prevented the evaluation of 6-MWT, DLCO, and PFT parameters over time, so disease progression and outcomes could not be studied. Third, owing to resource constraints, advanced imaging, including quantitative CT fibrosis scores, or serum biomarkers, including KL-6 and SP-D, were not used, and their correlation with functional parameters was not assessed. Finally, the effect of various factors, including comorbidities, medication use, and environmental exposures, on DLCO and 6-MWT was not assessed.

## Conclusions

In patients with ILD, the 6-MWT demonstrated a weak but significant and positive correlation with FVC, while correlation with FEV1 and DLCO was non-significant. Moreover, the correlation of DLCO with all PFT parameters remained non-significant. Significantly reduced actual 6-MWD compared to predicted 6-MWD suggests reduced tolerance to physical activity. These tests highlight functional limitations and physiological derangements, thus strengthening their role in ILD management. Further studies are required to explore the multi-dimensional predictors of exercise intolerance to optimize the care of patients with ILD.
